# ^18^F-FDG PET Metabolic Parameters and MRI Perfusion and Diffusion Parameters in Hepatocellular Carcinoma: A Preliminary Study

**DOI:** 10.1371/journal.pone.0071571

**Published:** 2013-08-05

**Authors:** Sung Jun Ahn, Mi-Suk Park, Kyung Ah Kim, Jun Yong Park, InSeong Kim, Won Joon Kang, Seung-Koo Lee, Myeong-Jin Kim

**Affiliations:** 1 Department of Radiology, Yonsei Liver Cancer Special Clinic, Yonsei University College of Medicine, Seoul, Republic of Korea; 2 Research Institute of Radiological Science, Yonsei Liver Cancer Special Clinic, Yonsei University College of Medicine, Seoul, Republic of Korea; 3 Division of Gastroenterology, Yonsei Liver Cancer Special Clinic, Yonsei University College of Medicine, Seoul, Republic of Korea; 4 Department of Nuclear Medicine, Yonsei Liver Cancer Special Clinic, Yonsei University College of Medicine, Seoul, Republic of Korea; 5 Siemens Medical System, Forchheim, Germany; The University of Chicago, United States of America

## Abstract

**Objectives:**

Glucose metabolism, perfusion, and water diffusion may have a relationship or affect each other in the same tumor. The understanding of their relationship could expand the knowledge of tumor characteristics and contribute to the field of oncologic imaging. The purpose of this study was to evaluate the relationships between metabolism, vasculature and cellularity of advanced hepatocellular carcinoma (HCC), using multimodality imaging such as ^18^F-FDG positron emission tomography (PET), dynamic contrast enhanced (DCE)-MRI, and diffusion weighted imaging(DWI).

**Materials and Methods:**

Twenty-one patients with advanced HCC underwent ^18^F-FDG PET, DCE-MRI, and DWI before treatment. Maximum standard uptake values (SUV_max_) from ^18^F-FDG-PET, variables of the volume transfer constant (K^trans^) from DCE-MRI and apparent diffusion coefficient (ADC) from DWI were obtained for the tumor and their relationships were examined by Spearman’s correlation analysis. The influence of portal vein thrombosis on SUV_max_ and variables of K^trans^ and ADC was evaluated by Mann-Whitney test.

**Results:**

SUV_max_ showed significant negative correlation with K^trans^_max_ (ρ = −0.622, p = 0.002). However, variables of ADC showed no relationship with variables of K^trans^ or SUV_max_ (p>0.05). Whether portal vein thrombosis was present or not did not influence the SUV _max_ and variables of ADC and K^trans^ (p>0.05).

**Conclusion:**

In this study, SUV was shown to be correlated with K_trans_ in advanced HCCs; the higher the glucose metabolism a tumor had, the lower the perfusion it had, which might help in guiding target therapy.

## Introduction

Hepatocellular carcinoma (HCC) is the third most common cause of cancer-related death globally, behind only lung and stomach cancer. Treatment options are limited for patients with advanced HCC and potentially curative treatments can be attempted in only 30–40% of patients. Conventional cytotoxic chemotherapy agents have not improved survival outcomes and standard treatment for advanced HCC has yet to be established. However, recently developed molecularly targeted agents offer new options for treating this chemo-resistant tumor, and have been reported to present survival benefits for advanced HCC [1], [2]. These molecular target agents are expensive and exert moderate side effects, so it is important to predict optimal candidates for this treatment. Because the therapeutic effects of these molecular agents usually depend on the proliferative and angiogenic activity of the tumor, it is important to understand these characteristics.

During the early stage of hepatocarcinogenesis, arterial blood supply increases as histologic grade progresses. However, tumor metabolism and angiogenesis in advanced HCC have not been well evaluated and could be different from those in early stage HCC, as well as affect treatment responses to the target agent.

^18^F-2-fluoro-2-deoxyglucose (18F-FDG) with positron emission tomography (PET), diffusion weighted MRI (DW-MRI), and dynamic contrast-enhanced MRI (DCE-MRI) provide the information of glucose metabolism, cellularity, and vascularity of the tumor [3]. Several studies have investigated the usefulness of functional imaging parameters in the prediction of a response in various tumors [4]–[9]. In previous studies regarding HCCs treated with antiangiogenic agents, lower SUV_max_ groups showed significantly longer overall survival than higher SUV_max_ groups and the changes of K^trans^ (a volume transfer constant) after treatment were correlated with the response to the antiangiogenic agent [5], [6], [8], [9].

We hypothesized that glucose metabolism, perfusion, and water diffusion may have a relationship or affect each other in the same tumor. The understanding of the functional imaging markers could expand the knowledge of tumor characteristics and allow for their wider clinical application in the fields of oncologic imaging. To our knowledge, however, no report has studied the relationships of the three parameters.

In this study, we investigated the relationships between tumor metabolism determined by ^18^F-FDG PET, tumor vasculature determined by DCE MRI, and tumor cellularity determined by diffusion MRI in patients with advanced hepatocellular carcinoma (HCC).

## Materials and Methods

The protocol for this retrospective study was approved by Severance Hospital, Institutional Review Board and informed consent for this retrospective study was not required. Our institutional review board waived the need for written informed consent from the participants.

One author is employed by Siemens Medical System (Forchheim, Germany). However, this does not alter our adherence to all the PLOS ONE policies on sharing data and materials. The other authors maintained full control of all data reported in this article at all times.

### Patients

By searching a database of prospectively collected data, we identified 26 patients with HCC who underwent PET-CT and DCE with DWI MRI within an interval of one month. Patients were excluded from the study if they underwent any treatment for HCC before MRI and PET scanning (n = 5). Finally, 21 patients (male: female, 17: 4; age range, 35–75 years; mean age, 56 years) were enrolled. Patients were classified as having advanced HCC if they were not eligible for surgical resection or locoregional therapies (stage C) according to the Barcelona Clinic Liver Cancer (BCLC) staging system [10].

### MR Protocol

All imaging studies were performed using a 3-T MR scanner (MAGNETOM Tim Trio; Siemens Healthcare, Erlangen, Germany) equipped with 8-channel body phased-array coils (Siemens Healthcare). The patients were asked to fast four hours before scanning. No antiperistaltic or oral contrast agents were used.

Coronal and axial T2WI HASTE images (TR/TE = 500/95 ms, number of slices = 20, thickness = 8 mm, field of view = 320 mm, matrix = 256×256) were acquired for localization.

Free-breathing DWI was performed with a singleshot, echo-planar sequence with motion-probing gradients in 3 directions(TR/TE = 6017/69 ms, field of view = 330×440 mm, matrix = 192×108, flip angle = 90, slice thickness = 5 mm, gap = 1 mm, number of slices = 30–40, number of excitation = 2, b values = 50, 400, and 800 second/mm^2^). After image acquisition, ADCs were automatically calculated by the MR system and displayed as corresponding ADC maps.

Dynamic contrast-enhanced MR imaging included two precontrast T1 weighted measurements (3D VIBE, TR/TE = 4.9/1.7 ms, field of view = 300×300 mm, matrix = 192×138, slice thickeness = 4 mm, number of slices = 20) with different flip angles (FAs) (2°, 15°) to determine the T1 relaxation time in the blood and tissue before contrast agent arrival on a pixel-by-pixel basis. This was followed by a dynamic contrast enhanced series using a Time-resolved angiography with interleaved stochastic trajectories sequence (TWIST, TR/TE = 4.5/1.7 ms, flip angle = 12°, temporal resolution = 0.295 s and all other parameters : same as precontrast image) after injecting 15 ml of Omniscan (gadodiamide; GE Healthcare, Oslo, Norway) at 5 ml/sec using an automatic injector, followed by a 30-ml saline injection. Serial images were acquired under shallow free-breathing conditions. FOV was focused on the center of tumor mass along the z axis. Perfusion images were acquired repetitively over 75 cycles for 7–8 min. On completion of the study, the data were transferred to an image processing workstation (Leonardo, Siemens Medical Solutions).

### PET Protocol

All patients fasted for more than six hrs before the procedure. They then signed informed consent for the procedure and received 5.5 MBq/kg of body weight of ^18^F- FDG intravenously over 2 min. After a 45-min equilibration period during which the patients were at rest, attenuation corrected emission images over the tumor were acquired on a PET-CT scanner, Biograph Truepoint 40 PET-CT (Siemens Medical Systems, CTI, Knoxville, TN, USA). Reconstructed attenuation corrected images were viewed in the transaxial, coronal, and sagittal planes.

### Image Analysis

**Figure 1:**
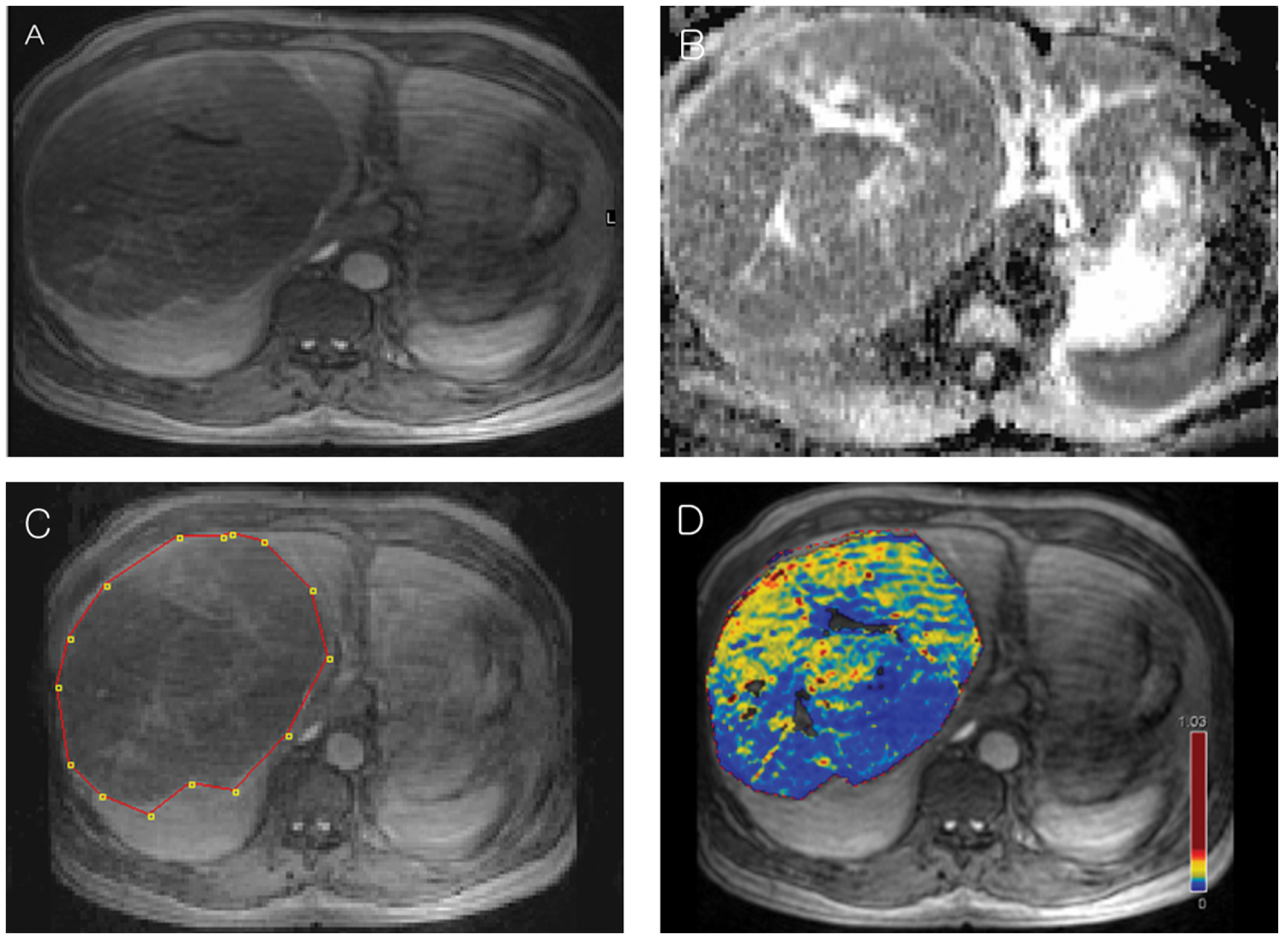
Advanced HCC in the Rt. lobe is well differentiated from normal liver parenchyma on delayed phase of DCE MRI(A), 7∼8 min after contrast injection. After ADC map (B) is coregistered to delayed phase of DCE MRI(A), ROI(red dotted line) was drawn along the tumor border on the coregistered image(C) and ADC value was calculated. K^trans^ color coded map(D) is also achieved based on the delayed phase of DCE MRI.

A third year resident and a board certified abdominal radiologist independently drew ROI (region of interest) on post-processed quantitative maps for calculation of ADC, K_trans_ and SUV. Their averaged values between two readers were used for further analysis. Detailed post processing methods are as follows;

ADC maps were co-registered to the last phase on DCE MRI (7∼8 minutes after contrast injection) using commercial software (nordicICE, NordicNeuroLab), based on Digital Imaging and Communication in Medicine geometry parameters (Figure 1). Manual adjustment of image registration was performed if necessary. ROI for ADC was drawn along the tumor border on the same ROIs as co-registered K^trans^ map. Maximum, minimum, median, and mean values of ADC (ADC_max_, ADC_min_, ADC_median_ and ADC_mean_) for the covered tumor volume were calculated. Tumors were distinctly differentiated from the normal parenchyma on this co-registered image.

Post-processing of K^trans^ from all series of the DCE MRI was performed with commercial software (tissue 4D, Siemens Medical Solutions) based on a modified Tofts model. Motion correction was performed on the dynamic images based on non-rigid registration technique [11] and a T1 map was registered to the dynamic images. After complete calculation of K^trans^, readers drew ROI along the tumor borders based on the last phase of DCE. Maximum, minimum, median and mean values of K^trans^ (K^trans^_max_, K^trans^_min_, K^trans^_median_, and K^trans^_mean_) for the covered tumor volume were calculated. The presence or absence of portal vein thrombosis was also recorded.

For SUV measurement, The 3D ROI was drawn to follow the contours of the elevated FDG activity as compared to the normal liver parenchyma. The maximal standardized uptake value (SUV) was calculated with the following formula: SUV = Cdc/(di/w), where Cdc was the decay-corrected tracer tissue concentration (in Becquerel per gram), di was the injected dose (in Becquerel), and w was the patient’s body weight (in grams). We defined SUV_max_ for each patient as the maximum measured SUV of the most hypermetabolic lesion.

### Statistical Analysis

Interobserver variability for the ADC, K^trans^, and SUV_max_ measurements between the two readers was analyzed by the intraclass correlation coefficient. The relationships among the matched quantitative imaging parameters (e.g., ADC_mean_ - K^trans^_mean_) from PET, DWI and DCE MRI images were examined by Spearman’s correlation analysis. In addition, the Mann-Whitney test was used to evaluate the influence of portal vein thrombosis on quantitative parameters of PET and MRI. A *P*-value of less than 0.05 was considered statistically significant. Statistical analyses were performed with Medcalc, version 9 (Medcalc Software, Belgium).

## Results

The averaged values of all the quantitative parameters for 21 patients were summarized in the Table 1. Intraclass correlation coefficients between two readers are 0.85 to 0.92, 0.82 to 0.95, and 0.99 for ADC, K^trans^ and SUV, respectively. The correlation between variables of ADC and K^trans^ are summarized in Table 2. There was no significant relationship between the corresponding parameters.

**Table 1:** Averaged values of ADC, K^trans^ and SUV of 21 patients with advanced HCC.

Quantitative parameters	Averaged value
ADC_max(x10_^−3^_mm_^2^_/s)_	2.854±0.561
ADC_min(x10_^−3^_mm_^2^_/s)_	0.091±0.132
ADC_median(x10_^−3^_mm_^2^_/s)_	1.472±0.123
ADC_mean(x10_^−3^_mm_^2^_/s)_	1.081±0.164
K^trans^_max(Sec_^−1^_)_	1.822±0.912
K^trans^_min(Sec_^−1^_)_	0.001±0.003
K^trans^_median(Sec_^−1^_)_	0.916±0.447
K^trans^_mean(Sec_^−1^_)_	0.143±0.071
SUV_max_	7.07±3.52
Size(mm)	106±36

Data are expressed as mean ± SD.

Tumor size indicates the maximum z axis diameter of tumor.

**Table 2:** The correlation between histogram measures of ADC and K^trans^.

	ADC_max_	ADC_min_	ADC_median_	ADC_mean_
K^trans^_max_	−0.213/0.352			
K^trans^_min_		0.212/0.355		
K^trans^_median_			−0.137/0.553	
K^trans^_mean_				0.145/0.529

Data are expressed as correlation coefficient(*ρ)/p-value.*

The correlations between SUV _max_ vs. K^trans^_max_ and SUV _max_ vs. ADC_max_ are summarized in Table 3. SUV_max_ showed a significant negative correlation with K^trans^_max_ (ρ = −0.622, p = 0.002). SUV_max_ showed no significant correlation with ADC_ max_ (ρ = 0.369, p = 0.099). Whether portal vein thrombosis was present or not did not influence any of the quantitative parameters: SUV _max_ (p = 0.756), ADC_min_ (p = 0.973), ADC_max_ (p = 0.282), ADC_mean_ (p = 0.251), ADC_median_ (p = 0.349) and K^trans^_min_ (p = 0.223), K^trans^_max_ (p = 0.918), K^trans^_mean_ (p = 0.756) and K^trans^_median_ (p = 0.863).

**Table 3:** The correlation between variables of ADC, K^trans^ and SUV_max._

	SUV_max_		SUV_max_
K^trans^_max_	−0.622/0.002*	ADC_max_	0.369/0.099

Data are expressed as correlation coefficient(*ρ)/p-value.*

The statistically significant correlations are indicated with an asterisk (*).

## Discussion

Our study revealed a significant negative correlation between SUV and K^trans^, indicating that advanced HCC with higher glucose metabolism tends to have lower perfusion. On the contrary, advanced HCC with lower glucose metabolism tends to have higher perfusion. Regarding vascularity in advanced HCC, our results were consistent with a previous study using perfusion CT, which reported that the perfusion values in poorly or moderately differentiated HCCs were lower than those in well-differentiated HCCs [12], [13]. These observations stand in contrast to the conventional idea that the higher grade a tumor is, the higher the vascularity it has. In the early stage of tumor angiogenesis, tumor vascularity may keep step with tumor growth. In the advanced stage, however, the growth of a tumor may leap ahead of the growth of vascularity, resulting in hypoperfusion or necrosis[14]–[17]. Low grade gliomas may have aerobic metabolic pathway meanwhile high grade glioma may have anaerobic metabolism [18]. Similarly, in early stages, HCCs may recruit arterial flow and use aerobic metabolism, then switch to anaerobic metabolism with less arterial supply at some point of moderately differentiated [19]. Our negative correlation between glucose metabolism and perfusion in advanced HCC may be explained on the base of increased anaerobic metabolism in advanced HCC [20]. Therefore, the target therapy in advanced HCC should be focused on the anaerobic metabolism rather than angiogenesis or vascularity. However, considering moderate correlation coefficient(ρ = 0.656∼0.660), each parameter may have independent domains that are not explained by the complex interplay between perfusion and glucose metabolism. Moreover, these independent domains might provide complementary information for therapy planning and response monitoring. Future studies may shed new light on these issues, as well as uncover detailed associations between functional imaging parameters.

According to our study, variables of ADC in advanced HCC showed no significant relationship with K^trans^ or SUV, indicating that diffusion is not correlated with perfusion or glucose metabolism; however, in addition to cellularity, ADC value is also influenced by necrosis or other factors.

Our study showed that the presence or absence of portal vein thrombosis did not influence perfusion, cellularity, and glucose metabolism in advanced HCCs. Our results were consistent with a previous study using perfusion CT, which showed that perfusion parameters were not different between patients with portal vein thrombosis and those without [12]. This observation could be explained by the hypothesis that HCCs are supplied by hepatic arteries but not by portal veins [12]. Therefore, portal vein thrombosis may not influence perfusion, cellularity and glucose metabolism in advanced HCCs.

Our study has several shortcomings. Firstly, there was a selection bias because only patients with advanced HCC were included. Secondly, we compared DWI with DCE MRI with different slice thickness and gap. There might be missing information of DWI to match with DCE. However, we used interpolation and co-registration method to overcome mismatches [21], [22]. Thirdly, we did not perform pathologic examination. Detailed associations between functional imaging parameters should be supported by pathologic examination in future studies.

In this study, SUV was shown to be correlated with K_trans_ in advanced HCCs; the higher the glucose metabolism a tumor had, the lower the perfusion it had, which might help in guiding target therapy.
